# Fluorescent markers of the microtubule cytoskeleton in *Zymoseptoria tritici*

**DOI:** 10.1016/j.fgb.2015.03.005

**Published:** 2015-06

**Authors:** M. Schuster, S. Kilaru, M. Latz, G. Steinberg

**Affiliations:** Biosciences, University of Exeter, Exeter EX4 4QD, UK

**Keywords:** Tub2, α-tubulin, Zt, *Zymoseptoria tritici*, Peb1, homologue of the mammalian plus-end binding protein EB1, Grc1, component of the minus-end located γ-tubulin ring complex, Tub1, beta-tubulin, *sdi1*, succinate dehydrogenase 1, RB and LB, right and left border, eGFP, enhanced green fluorescent protein, mCherry, monomeric cherry, *hph*, hygromycin phosphotransferase, *nptII*, neomycin phosphotransferase, *bar*, phosphinothricin acetyltransferase, Microtubules, Mitosis, Wheat pathogenic fungi, Septoria tritici blotch, *Mycosphaerella graminicola*

## Abstract

The microtubule cytoskeleton supports vital processes in fungal cells, including hyphal growth and mitosis. Consequently, it is a target for fungicides, such as benomyl. The use of fluorescent fusion proteins to illuminate microtubules and microtubule-associated proteins has led to a break-through in our understanding of their dynamics and function in fungal cells. Here, we introduce fluorescent markers to visualize microtubules and accessory proteins in the wheat pathogen *Zymoseptoria tritici*. We fused enhanced green-fluorescent protein to α-tubulin (ZtTub2), to ZtPeb1, a homologue of the mammalian plus-end binding protein EB1, and to ZtGrc1, a component of the minus-end located γ-tubulin ring complex, involved in the nucleation of microtubules. *In vivo* observation confirms the localization and dynamic behaviour of all three markers. These marker proteins are useful tools for understanding the organization and importance of the microtubule cytoskeleton in *Z. tritici*.

## Highlights

• We establish *Z. tritici* markers to visualize microtubules and their plus- and minus-ends.
• All markers localize correctly, determined by co-localization and pharmaceutical experiments.
• We provide 4 carboxin-resistance conveying vectors for targeted integration into the *sdi1*, *eb1* or *grc1* locus.
• We provide 3 hygromycin B-resistance conveying vectors for integration into the *eb1* or *grc1* locus or for random integration.

## 1 Introduction

Microtubules are an essential part of the eukaryotic cytoskeleton. They are dynamic polymers that consist of α- and β-tubulin subunits (Desai and Mitchison, 1997). Microtubules are formed by γ-tubulin (Oakley and Oakley, 1989) at microtubule-organizing centres, where a γ-tubulin ring complex nucleates the polymerization of the tubulin dimers (Job et al., 2003; Moritz et al., 1995; Teixido-Travesa et al., 2012; Zheng et al., 1991). The core of this complex is formed by three proteins, that is, γ-tubulin, Spc97- and Spc98-like homologues, which are conserved from fungi to humans (Knop and Schiebel, 1997; Riehlman et al., 2013; Vardy and Toda, 2000), overview in Wiese and Zheng (2006). In the filamentous fungus *Ustilago maydis*, γ-tubulin and the Spc97-homologue Grc1 localize to the nuclear spindle pole body (Straube et al., 2003), which nucleate microtubules in mitosis, but also to cytoplasmic nucleation sites (Fink and Steinberg, 2006; Schuster et al., 2011).

Microtubules play an essential role in fungi. They are a pivotal part of the mitotic spindle and are required for chromosome segregation during the mitotic phase (Fink et al., 2006; Horio et al., 1991; Martin et al., 1997). During interphase, microtubules form long and often stable “tracks”. These tracks are used by associated molecular motors to deliver secretory vesicles, move organelles, mRNA particles, ribosomes and effector proteins (Abenza et al., 2009; Baumann et al., 2012; Bielska et al., 2014; Higuchi et al., 2014; Schuster et al., 2012; Wedlich-Söldner et al., 2000) overview in Egan et al. (2012), Steinberg (2014), Xiang and Plamann (2003). Two classes of motors utilize microtubules; most kinesins “walk” from tubulin dimer to dimer towards the plus-end, whereas dynein takes cargo to the minus-end of microtubules (Hirokawa and Takemura, 2004; Vale, 2003). In many fungi, microtubule plus-ends are directed towards the growing hyphal tip, suggesting that microtubule-dependent transport supports hyphal growth. Indeed, microtubules and associated kinesin motors were shown to support hyphal growth in *U. maydis*, *Aspergillus nidulans* and *Neurospora crassa* (Fuchs et al., 2005; Horio and Oakley, 2005; Konzack et al., 2005; Lenz et al., 2006). Microtubules support the delivery of secretory vesicles (Schuchardt et al., 2005; Schuster et al., 2012; Pantazopoulou et al., 2014), but also provide tracks for motility of early endosomes, which are transported by kinesin motors in *U. maydis* (Wedlich-Söldner et al., 2002), *A. nidulans* (Abenza et al., 2009) and *N. crassa* (Seidel et al., 2013). Interestingly, studies in *U. maydis* revealed that kinesin-3 also supports transport of early endosomes away from the hyphal tip. This bi-directional transport of a uni-directional motor is possible due to an anti-polar microtubule orientation (plus-ends are directed towards the hyphal tip and towards the centre of the cell; Schuster et al., 2011). Thus, knowing the directionality of microtubules is pivotal to understand the molecular machinery behind a transport process. Due to the essential roles of microtubules in filamentous fungi, anti-microtubule fungicides have been used widely to control fungal diseases (Clemons and Sisler, 1971, overview in Holloman et al., 1997). Here, we establish fluorescent marker proteins for investigation of microtubule organization and dynamics in the wheat pathogen *Zymoseptoria tritici*.

## 2 Materials and methods

### 2.1 Bacterial and fungal strains and growth conditions

*Escherichia coli* strain DH5α was used for the maintenance of plasmids. *Agrobacterium tumefaciens* strain EHA105 (Hood et al., 1993) was used for maintenance of plasmids and subsequently for *A. tumefaciens*-mediated transformation of *Z. tritici. E. coli* and *A. tumefaciens* were grown in DYT media (tryptone, 16 g/l; yeast extract, 10 g/l; NaCl, 5 g/l; with 20 g/l agar added for preparing the plates) at 37 °C and 28 °C respectively. The fully sequenced *Z. tritici* wild-type isolate IPO323 (Goodwin et al., 2011; Kema and van Silfhout, 1997) was used as recipient strain for the genetic transformation experiments. Cells were maintained as glycerol stocks (NSY glycerol; nutrient broth, 8 g/l; yeast extract, 1 g/l; sucrose, 5 g/l; glycerol, 700 ml/l), and cultures were grown on YPD agar (yeast extract, 10 g/l; peptone, 20 g/l; glucose, 20 g/l; agar, 20 g/l) at 18 °C for 4–5 days.

### 2.2 Identification of *Z. tritici* homologues and bioinformatics

To identify homologues of the chosen marker proteins, we screened the published genome sequence of *Z. tritici* strain IPO323 (http://genome.jgi.doe.gov/Mycgr3/Mycgr3.home.html) using protein BLAST (http://blast.ncbi.nlm.nih.gov/Blast.cgi) and the *U. maydis* proteins sequences of Tub1 (accession number: XP_757368.1), Peb1 (Straube et al., 2003; accession number: XP_761908.1) and Grc1 (Schuster et al., 2011; accession number: XP_757621.1). Sequences were obtained from the NCBI server (http://www.ncbi.nlm.nih.gov/pubmed) and comparisons were done using CLUSTAL W (http://www.ebi.ac.uk/Tools/msa/clustalw2/) and EMBOSS Needle (http://www.ebi.ac.uk/Tools/psa/emboss_needle/). This revealed that a mistake in the annotation of ZtGrc1, which is predicted without a stop codon. We choose the next down-stream stop codon as the end of the open reading frame. This extended the predicted protein length by 10 amino acids. This corrected ZtGrc1 sequence was used for further analyzis and cloning. Domain structures were analyzed in PFAM (http://pfam.xfam.org/search/sequence). Finally, phylogenetic trees were generated in MEGA5.2, using Neighbor-Joining algorithm, followed by 1000 bootstrap cycles (http://www.megasoftware.net/; Tamura et al., 2011).

### 2.3 Molecular cloning

All the vectors used in this study were generated by *in vivo* recombination in the yeast *Saccharomyces cerevisiae* DS94 (MATα, *ura3-52*, *trp1-1*, *leu2-3*, *his3-111*, and *lys2-*801 (Tang et al., 1996), following published procedures (Raymond et al., 1999; Kilaru et al., 2015b). For all the recombination events, the fragments were amplified with 30 bp homologous sequences to the upstream and downstream of the fragments to be cloned (see Table 1 for primer details). PCR reactions and other molecular techniques followed standard protocols (Sambrook and Russell, 2001). The DNA fragments of interest were excised from the agarose gel and purified by using silica glass suspension as described previously (Boyle and Lew, 1995). Plasmid DNA was isolated from the positive yeast colonies as described previously (Hoffman and Winston, 1987). All restriction enzymes and reagents were obtained from New England Biolabs Inc. (NEB, Herts, UK).

### 2.4 Vectors to visualize microtubules

The vector pCeGFPTub2 contains *egfp* fused to full-length *Z. tritici tub2* gene under the control of *Z. tritici tub2* promoter for integration in to the *sdi1* locus by using carboxin as selection agent. A 9760 bp fragment of pCGEN-YR (digested with *Xba*I and *Zra*I), 706 bp of exon 3 of *sdi1* gene (amplified with SK-Sep-10 and SK-Sep-11; Table 1), a point-mutated (H267L) 308 bp fragment covering the last 111 bp of 3′ end *sdi1* gene and 197 bp downstream of the *sdi1* gene (amplified with SK-Sep-12 and SK-Sep-13; Table 1), 1149 bp *Z. trirtici tub2* promoter (amplified with SK-Sep-14 and SK-Sep-15; Table 1), 717 bp *egfp* (amplified with SK-Sep-16 and SK-Sep-17; Table 1), 1688 bp full length *tub2* gene and 1086 bp *tub2* terminator (amplified with SK-Sep-18 and SK-Sep-19; Table 1; Table 1) and 889 bp covering the right flank of *sdi1* gene (amplified with SK-Sep-25 and SK-Sep-26; Table 1) were recombined in yeast *S. cerevisiae* to obtain the vector pCeGFPTub2 (Fig. 2A).

The vector pCmCherryTub2 contains *mCherry* fused to full-length *Z. tritici tub2* gene under the control of *Z. tritici tub2* promoter for integration in to the *sdi1* locus by using carboxin as selection agent. A 12,530 fragment of pCeGFPTub2 (Fig. 2A; digested with *Bsr*GI), 1149 bp *Z. tritici tub2* promoter (amplified with SK-Sep-14 and SK-Sep-47; Table 1), 708 bp *mCherry* (amplified with SK-Sep-83 and SK-Sep-92; Table 1), 1688 bp full-length *tub2* gene and 1086 bp *tub2* terminator (amplified with SK-Sep-18 and SK-Sep-19; Table 1) were recombined in yeast *S. cerevisiae* to obtain the vector pCmCherryTub2 (Fig. 2D).

This vector pHeGFPTub2 contains *egfp* fused to full-length *Z. tritici tub2* gene under the control of *Z. tritici tub2* promoter for random ectopic integration using hygromycin B as selection agent. A 15,219 bp fragment of pCeGFPTub2 (Fig. 2A; digested by *Bgl*II *and Bam*HI) and 1510 bp hygromycin resistance cassette (amplified with SK-Sep-128 and SK-Sep-129; Table 1) were recombined in yeast *S. cerevisiae* to obtain the vector pHeGFPTub2 (Fig. 2E). Note that this vector was derived from carboxin resistance conferring vector pCeGFPTub2 (Fig. 2A) and as such it contain part of the succinate dehydrogenase gene, carrying the mutation H267L and succinate dehydrogenase terminator. However, these fragments are of no significance.

### 2.5 Vectors to visualize microtubule-plus ends

Vector pCEB1eGFP contains *egfp* fused to partial *Z. tritici eb1* gene for targeted integration in to the *eb1* locus of *Z. tritici* using carboxin as selection agent. A 9533 bp fragment of pCeGFPTub2 (Fig. 2A; digested with *Bam*HI and *Hind*III), 3′ end of 686 bp *eb1* (without stop codon; amplified with SK-Sep-48 and SK-Sep-49; Table 1), 720 bp *egfp* and 304 bp *Tnos* terminator (amplified with SK-Sep-16 and SK-Sep-60; Table 1), 1027 bp of *sdi1* promoter and 897 bp (5′ end of the gene) of H267L mutated, carboxin-resistance conferring allele of *sdi1* gene (amplified with SK-Sep-96 and SK-Sep-11; Table 1), 111 bp (3′ end of the gene) of H267L mutated allele of *sdi1* gene and 197 bp of *sdi1* terminator (amplified with SK-Sep-12 and SK-Sep-97; Table 1) and 681 bp *eb1* right flank covering the downstream of the *eb1* gene, immediately after the stop codon (amplified with SK-Sep-100 and SK-Sep-51; Table 1) were recombined in yeast *S. cerevisiae* to obtain the vector pCEB1eGFP (Fig. 2F).

Vector pHEB1eGFP is identical to the vector pCEB1eGFP, but contains a hygromycin resistance cassette instead of carboxin resistance cassette. A 13,117 bp fragment of vector pCEB1eGFP (Fig. 2F; digested with *ZraI* and *Hind*III) and a 1510 bp hygromycin resistant cassette (amplified with SK-Sep-130 and SK-Sep-133; Table 1) were recombined in yeast *S. cerevisiae* to obtain the vector pHEB1eGFP (Fig. 2B).

### 2.6 Vectors to visualize microtubule-minus ends

Vector pCGrc1eGFP contains *egfp* fused to partial *Z. tritici grc1* gene for targeted integration in to the *grc1* locus of *Z. tritici* using carboxin as selection agent. A 9533 bp fragment of pCeGFPTub2 (Fig. 2A; digested with *Bam*HI and *Hind*III), 3′ end of 1049 bp *grc1* (without stop codon; amplified with SK-Sep-37 and SK-Sep-38; Table 1), 720 bp *egfp* and 304 bp *Tnos* terminator (amplified with SK-Sep-16 and SK-Sep-60; Table 1), 1027 bp of *sdi1* promoter and 897 bp (5′ end of the gene) of H267L mutated allele of *sdi1* gene (amplified with SK-Sep-96 and SK-Sep-11; Table 1), 111 bp (3′ end of the gene) of H267L mutated, carboxin-resistance conferring allele of *sdi1* gene and 197 bp of *sdi1* terminator (amplified with SK-Sep-12 and SK-Sep-97; Table 1) and 1027 bp *grc1* right flank covering the downstream of the *grc1* gene, immediately after the stop codon (amplified with SK-Sep-99 and SK-Sep-53; Table 1) were recombined in yeast *S. cerevisiae* to obtain the vector pCGrc1eGFP (Fig. 2 F).

Vector pHGrc1eGFP is identical to the vector pCGrc1eGFP, but contains a hygromycin resistance cassette instead of carboxin resistance cassette. A 13,686 bp fragment of vector pCGrc1eGFP (Fig. 2F; digested with *Bgl*II and *Sal*I) and a 1510 bp hygromycin resistant cassette (amplified with SK-Sep-130 and SK-Sep-132; Table 1) were recombined in yeast *S. cerevisiae* to obtain the vector pHGrc1eGFP (Fig. 2B). Further details on vector construction and yeast recombination-based cloning is provided in Kilaru and Steinberg (2015).

### 2.7 *Z*. *tritici* transformation and molecular analyzis of transformants

The vectors pCeGFPTub2, pCmCherryTub2, pHEB1eGFP and pGrc1eGFPwere transformed into *A. tumefaciens* strain EHA105 by heat shock method (Holsters et al., 1978) and *A. tumefaciens* mediated transformation *of Z. tritici* was performed as described previously by Zwiers and De Waard (2001) with the slight modifications. Further details on this method are provided in Kilaru et al. (2015a). The genomic DNA was purified from the transformants which exhibited green fluorescence and also the wild-type isolate IPO323. To confirm the integration of vector in to the *sdi1, eb1 and grc1* loci of *Z. tritici* and also to determine the copy number, Southern blot hybridizations were performed by using the standard procedures (Sambrook and Russell, 2001). 3 μg of genomic DNA of IPO323 and transformants obtained with various vectors were digested with *Bgl*II (for pCeGFPTub2 transformants), *Pvu*II (for pHEB1eGFP transformants) and *Xmn*I (for pHGRC1eGFP transformants) and separated on a 1.0% agarose gel and capillary transferred to a Hybond N^+^ membrane (Amersham Pharmacia Biotech). 1014 bp *sdi1* probe (3′ end of the *sdi1^R^* gene and *sdi1* terminator) was generated by with primers SK-Sep-10 and SK-Sep-13; Table 1 and 681 bp *eb1* probe (downstream non coding sequence of the *eb1* gene) was generated with primers SK-Sep-100 and SK-Sep-51; Table 1 and 1049 bp *grc1* probe (3′ end of the *grc1* gene) was generated with primers SK-Sep-37 and SK-Sep-38; Table 1. All the probes were generated by using DIG labelling PCR mix (Life Science Technologies, Paisley, UK). Hybridizations were performed at 62 °C for overnight and autoradiographs were developed after an appropriate time period.

### 2.8 Microscopy

Fluorescence microscopy was performed as described in Kilaru et al. (2015b). Microtubules were visualized by generating z-stacks with a *z* resolution of 0.2 μm and an exposure time of 150 ms. The 488 nm laser was used at 100%. The final images are maximum projections generated in MetaMorph (Molecular Devices, Wokingham, UK). The plus-end marker EB1 was imaged in time laps movies with an exposure time of 250 ms and a time interval of 500 ms. The 488 nm laser was used at 75%. To co visualize the microtubules in red and the plus end marker in green, z-stacks with a z resolution of 0.2 μm and an exposure time of 250 ms were taken. The 488 nm laser was used at 75% and the 561 nm laser was used at 100% output power. The image were 2D deconvolved followed be generating a maximum projection using MetaMorph (Molecular Devices, Wokingham, UK). The microtubules were depolymerised in the strains IPO323_CeGFPTub2 and IPO323_HEB1eGFP by incubating the cells in YG media containing 300 μM benomyl (Sigma–Aldrich Chemie GmbH, Munich, Germany) for 45 min at 18 °C with 200 rpm. Treated cells were placed onto a 2% agar cushion containing 300 μM benomyl (Sigma–Aldrich Chemie GmbH, Munich, Germany) and directly imaged using 150 ms exposure time and the 488 nm laser at 100%. To visualize the Grc1 at the spindle pole bodies Grc1-eGFP was co-localized with mitotic spindles labelled with mCherry-Tub2 by generating z-stacks with a z resolution of 0.2 μm and an exposure time of 150 ms. The 488 nm laser was used at 40% and the 561 nm laser was used at 75% output power. The final images are maximum projections generated in MetaMorph (Molecular Devices, Wokingham, UK).

## 3 Results and discussion

### 3.1 Identification of ZtTub2, ZtEB1 and ZtGrc1

**Fig. 1:**
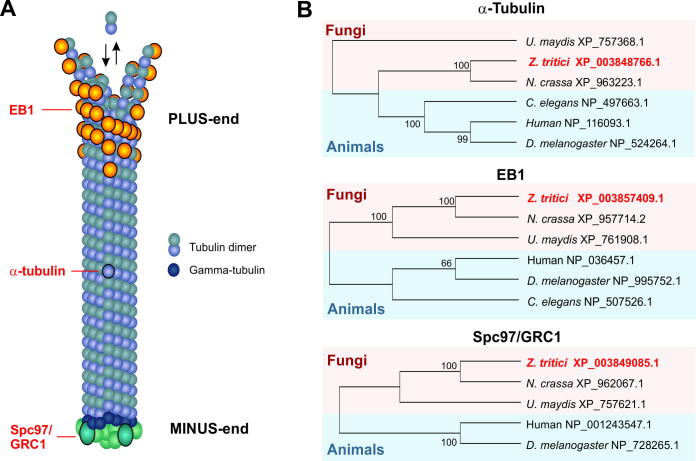
Marker proteins to visualize microtubules. (A) Diagram depicting the localization of microtubule marker proteins used in this study. The plus-end binding protein EB1 binds to the dynamic end of microtubules, where most polymerization and depolymerization of tubulin dimmers occur (indicated by arrows). As part of the tubulin dimer, α tubulin is incorporated into the microtubule lattice. The minus-end is linked to the γ-tubulin ring complex, which nucleates microtubules and consists of several proteins, including Spc97/GRC1. (B) Phylogenetic trees comparing the predicted full-length amino acid sequence of α tubulins, EB1-like proteins and Spc97/GRC1 homologues in animals (human, the fruit fly *Drosophila melanogaster*) and fungi (the ascomycetes *Neurospora crassa* and *Z. tritici* and the basidiomycete *Ustilago maydis*). The *Z. triti*ci orthologues, used in this study, are indicated in bold and red. NCBI accession numbers are given behind species names (http://www.ncbi.nlm.nih.gov/pubmed). Trees were generated using MEGA5.1 (Tamura et al., 2011). Bootstrap values are indicated at branching points. NCBI accession numbers are indicated.

In this study we set out to establish fluorescent marker proteins for microtubules and their plus- and minus-ends. We chose α-tubulin, an EB1-like plus-end binding protein and a γ-tubulin ring complex protein (Fig. 1A). We identified *Z*. *tritici* homologues of the *U. maydis* α-Tubulin, Tub1 (Steinberg et al., 2001), the EB1like protein Peb1 (Straube et al., 2003) and the γ-tubulin ring complex protein Grc1 (Schuster et al., 2011) by performing a BLASTP search with the *U. maydis* sequences against the *Z. tritici* published genome sequence of IPO323 at the Joint Genome Institute (http://genome.jgi.doe.gov/Mycgr3/Mycgr3.home.html). This revealed the putative α-tubulin (protein ID 76039; NCBI accession number: XP_003848766.1), which is 99.8% identical to the described α-tubulin from a different strain of *Z. tritici* (former *Septoria tritici*; Rohel et al., 1998; NCBI accession number: Y14509.1). As the beta-tubulin from *Z. tritici* was introduced as Tub1 (Bowler et al., 2010), we named the identified IPO323 α-tubulin as Tub2. Our bioinformatic approach also revealed an EB1-like protein, ZtEB1 (protein ID 65966; NCBI accession number: XP_003857409.1) and a putative microtubule minus-end-binding protein, ZtGrc1 (protein ID 63277; NCBI accession number: XP_003849085.1). A phylogenetic sequence comparison with homologues in other fungi and in animal cells confirms the predicted identity of all three putative markers (Fig. 1B). The proteins ZtTub2 and ZtEB1 share significant amino acid sequence identity with their counterparts in *U. maydis* (UmTub1/ZtTub2 = 77.4%*,* UmPep1/ZtEB1 = 47.3%). In addition, ZtTub2 and ZtEB1 share domains with their counterparts in *U. maydis* (ZtTub2: Tubulin/FtsZ family, GTPase domain, *P* = 2.7e−68, Tubulin C-terminal domain, *P* = 6.4e−49; ZtEB1: Calponin homology domain, *P* = 4.2e−05, EB1-like C-terminal motif, *P* = 6.3e−17). In contrast, the putative γ-tubulin ring complex protein ZtGrc1 shared only 17.6% amino acid sequence identity with UmGrc1, 18.8% with Spc97 from *S. cerevisiae* (NCBI accession number: EGA58404.1) and 17% with Spc98 from *S. cerevisiae* (NCBI accession number: EEU08751.1). However, ZtGrc1 contains a Spc97/Spc98 family domain (*P* = 3.5e−133). Therefore, we felt confident in establishing these *Z. tritici* as markers for microtubules and their plus- and minus end.

### 3.2 Vector for targeted ectopic integration of eGFP-Tub2 fusion construct

**Fig. 2:**
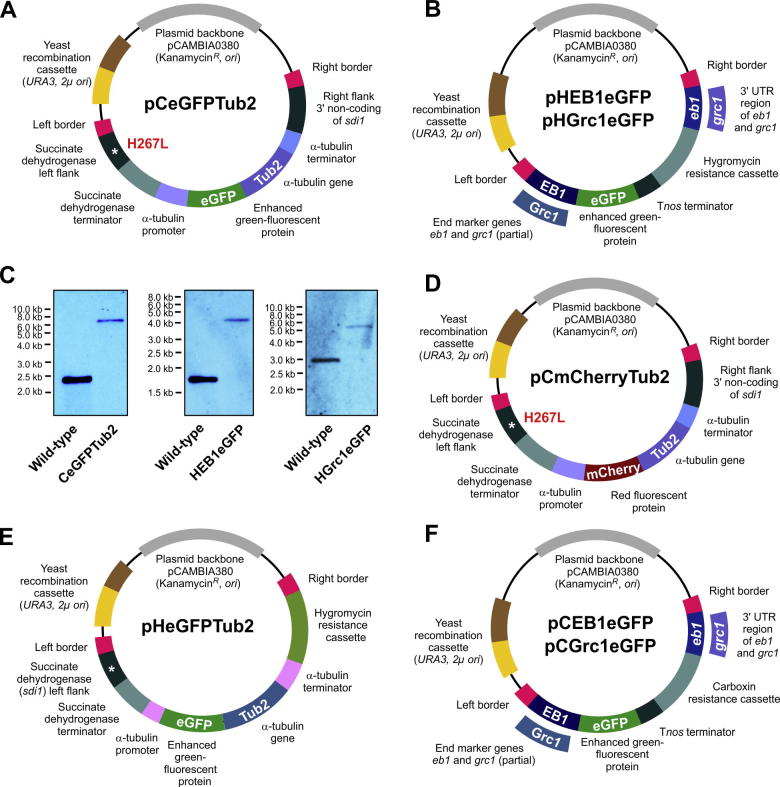
Vectors for integration of microtubule marker proteins in to the genome of *Z. tritici*. (A) Schematic drawing showing the organization of vector pCeGFPTub2. This vector contain the H267L point mutation in a stretch of *sdi1* sequence and after integration into the *sdi1* locus of *Z. tritici*, this mutation confers resistance to the anti-fungal drug carboxin (see Kilaru et al., 2015a, for more information). *tub2* is the α tubulin gene of *Z. tritici*, and the fluorescent protein eGFP-Tub2 is integrated into the lattice of microtubules. Note that fragments are not drawn to scale. For more accurate information on fragment sizes see main text. (B) Schematic drawing showing the organization of vectors pHEB1eGFP and pHGrc1eGFP. Both vectors allow integration of *egfp* into the native loci of *Z. tritici eb1* and *grc1* homologues using hygromycin B as selection agent. The fusion proteins label the plus- and minus-end of microtubules (see Fig. 1A). Note that fragments are not drawn to scale. For more accurate information on fragment sizes see main text. (C) Southern blots showing integration of pCeGFPTub2, pHEB1eGFP and pHGrc1eGFP into the *sdi1*, *eb1* and *grc1* locus of *Z. tritici* IPO323. The blot was hybridised with *sdi1* left flank, EB1 right flank and Grc1 left flank, respectively. After digestion of the genomic DNA with *Bgl*II, *Pvu*II, *Xmn*I, and subsequent hybridisation with corresponding labelled DNA probes, shifts in size of the signal from 2.3 kb, 1.7 kb and 2.9 kb to 7.0 kb, 4.2 kb and 5.4 kb, respectively. The size of DNA markers is indicated at the left; note that gels were run differently and marker sizes are therefore not comparable. (D) Schematic drawing showing the organization of vector pCemCherryTub2. This vector contain the H267L point mutation in a stretch of *sdi1* sequence and after integration into the *sdi1* locus of *Z. tritici*, this mutation confers resistance to the anti-fungal drug carboxin (see Kilaru et al., 2015a, for more information). *tub2* is the α tubulin gene of *Z. tritici*, and the fluorescent protein mCherry-Tub2 is integrated into the lattice of microtubules. Note that fragments are not drawn to scale. For more accurate information on fragment sizes see main text. (E) Schematic drawing showing the organization of vector pHeGFPTub2. The vector carries a hygromycin resistance cassette and is designed to enable random integration into the genome of *Z. tritici*. Note that fragments are not drawn to scale. For more accurate information on fragment sizes see main text. Note that this vector was derived from carboxin resistance conferring vector (Fig. 2A). As such it contains part of the succinate dehydrogenase gene, carrying the mutation H267L and succinate dehydrogenase terminator. However, these fragments are of no significance. (F) Schematic drawing showing the organization of vectors pCEB1eGFP and pCGrc1eGFP. Both vectors allow integration of *egfp* into the native loci of *Z. tritici eb1* and *grc1* homologues using carboxin as selection agent. The fusion proteins label the plus- and minus-end of microtubules (see Fig. 1A). Note that fragments are not drawn to scale. For more accurate information on fragment sizes see main text.

In order to visualize microtubules in *Z. tritici*, we constructed firstly the vector pCeGFPTub2 that allows the expression of eGFP-Tub2 fusion protein under the control of the constitutive *Z. tritici* α-tubulin (*tub2*) promoter (Fig. 2A). The vector was built into the *Agrobacterium* binary vector pCAMBIA0380 (CAMBIA, Canberra, Australia). It allows *A. tumefaciens*-based transformation into *Z. tritici*, which is based on the 25 bp imperfect directional repeat sequences of the T-DNA borders (right and left border, RB and LB; Fig. 2A). The vector also carries a kanamycin resistance gene and origins of replication for amplification in *E. coli* and *A. tumefaciens*. We designed this vector for targeted integration into the genomic *sdi1* locus of *Z. tritici*, by using a mutated downstream stretch of the *sdi1* sequence, carrying a carboxin resistance conferring point mutation (H267L; Fig. 2A, left flank), and a sequence stretch downstream of *sdi1* (Fig. 2A, right flank of *sdi1*). Incorporation by homologous recombination mutates the *sdi1* gene and integrates the eGFP-Tub2 construct into the *sdi1* locus (for details see Kilaru et al., 2015a). This result in single integration of the fusion construct without affecting other *Z. tritici* genes. In addition, this vector comprises a “yeast recombination cassette”, consisting of URA3 and 2μ *ori*, which enables yeast recombination-based cloning (for more details see Kilaru and Steinberg, 2015). All the further vectors, described in this study, were derived from the *Agrobacterium* binary vector pCAMBIA0380 and hence share these features.

### 3.3 Vectors containing EB1-eGFP and Grc1-eGFP fusion constructs for targeted integration in to the *eb1* and *grc1* loci

In corn smut fungus, microtubule plus-ends and minus-ends were visualized by fusing fluorescent proteins to the 3-prime end of the endogenous genes *eb1* and *grc1* respectively (Schuster et al., 2011; Straube et al., 2003). To visualize the homologues ZtEB1 and ZtGrc1 in *Z. tritici*, we constructed two vectors pHEB1eGFP and pHGrc1eGFP. Both vectors are designed to integrate the gene enhanced green fluorescent protein (*eGFP*) to the genomic loci of both genes, which ensures native expression levels of the fluorescent proteins ZtEB1-eGFP and ZtGrc1-eGFP. During homologous recombination in *Z. tritici*, the partial 3-prime sequences of the open-reading frame of *eb1* and *grc1* serve as left flanks, whereas 3′ non-coding regions of *eb1* and *grc1* serve as right flanks (Fig. 2B, see material and methods for details). The successful homologous recombination integrates the eGFP and the hygromycin resistance cassette into the genome.

### 3.4 Generation of *Z. tritici* strains with fluorescently labelled microtubules

In order to visualize microtubules, their plus- and minus-ends, vector pCeGFPTub2, pHEB1eGFP and pHGrc1eGFP were transformed into *Z. tritici* strain IPO323 (Kema and van Silfhout, 1997) using *A. tumefaciens* -mediated transformation (Zwiers and De Waard, 2001). Hygromycin B or carboxin-resistant transformants were screened for the presence of fluorescent signals. Correct integration of the fusion constructs to either the *sdi1* locus (Fig. 2C, pCeGFPTub2) or the native *eb1* and *grc1* loci of *Z. tritici* (Fig. 2C, pHEB1eGFP and pHGrc1eGFP) was confirmed by hybridising DNA from these transformants with gene specific probes (see materials and methods for details). In all cases, a single band was detected that appeared at the expected fragment sizes (7.0 kb, 4.2 kb and 5.4 kb; Fig. 2C). This confirmed that all constructs were correctly integrated as single copies. The resultant strains were named as IPO323_CeGFPTub2, IPO323_HEB1eGFP and IPO323_HGrc1eGFP, respectively.

### 3.5 A vector for targeted ectopic integration of a mCherry-Tub2 fusion construct

We next set out to enable the co-visualization of microtubules and the plus- and minus-end marker ZtEB1 and ZtGrc1, respectively. This set of experiments was designed to confirm that both markers indeed label microtubule ends. We generated vector pCmCherryTub2 that allows the expression of mCherry-Tub2 fusion protein under the control of constitutive *Z. tritici* α-tubulin (*tub2*) promoter (Fig. 2D). Again, this vector was designed for targeted integration into the genomic *sdi1* locus of *Z. tritici* (see explanation above; Fig. 2D). We transformed pCmCherryTub2 into the *Z. tritici* strains IPO323_HEB1eGFP and IPO323_HGrc1eGFP by using *A. tumefaciens* mediated transformation, and obtained the strains IPO323_HEB1eGFP_CmCherryTub2 and IPO323_HGrc1eGFP_CmCherryTub2.

### 3.6 Visualization of fluorescently labelled cytoskeletal components in *Z. tritici*

**Fig. 3:**
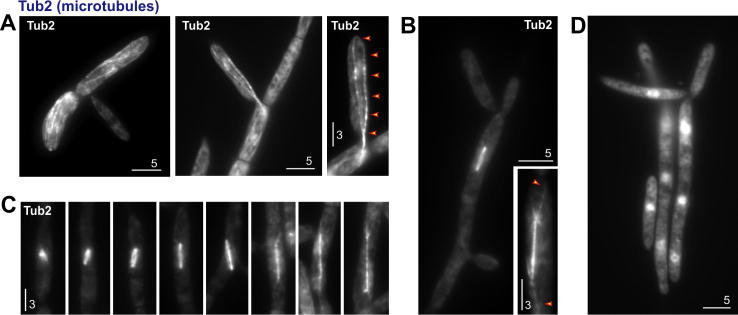
Microtubules in *Z. tritici*. (A) After integration of pCeGFPTub2, the fluorescent α tubulin is incorporated into microtubules. Microtubules reach through a bud-neck into the growing cell (middle panel). A maximum projection of a *Z*-axis stack of images reveals that microtubules form continuous tracks that reach to the tip of the growing cell (right panel, arrowheads). This suggests a role of microtubules in secretion in *Z. tritici*. Bar represents 5 and 3 μm. (B) eGFP-Tub2 allows visualization of mitotic spindles. The central spindle consists of numerous microtubules and, therefore, shows higher signal intensity than the astral microtubules (inset, arrowheads). Bar represents 5 and 3 μm. (C) Examples of mitotic spindles at different stages of elongation. Bar represents 3 μm. (D) Cells treated with the microtubule disrupting drug benomyl. Note that eGFP-ZtTub2 mainly localizes to the nuclei. Bar represents 5 μm.

α-tubulin forms part of the microtubule lattice (Fig. 1A) and was shown to integrate along the length of fungal microtubules (Drummond and Cross, 2000; Steinberg et al., 2001; Szewczyk and Oakley, 2011). We confirmed the localization of fluorescent ZtTub2 in strain IPO323_CeGFPTub2, which also expressed an untagged endogenous α-tubulin. We found long filamentous structures (Fig. 3A), which connected the growth region with the subapical parts of the cell (Fig. 3A, right panel, arrowheads; note that this figure is a maximum intensity projection of several planes, see Methods). The presence of untagged Tub2 may be responsible for the variations of the fluorescence along the length of the microtubules. eGFP-Tub2 was also incorporated into mitotic spindles (Fig. 3B and C), which consisted of a strong central bundle and much fainter astral microtubules, emanating from the spindle poles (Fig. 3B, arrowheads in inset). This appearance is reminiscent of mitotic spindle microtubules in *U. maydis* (Fink et al., 2006; Steinberg et al., 2001) and *A. nidulans* (Jung et al., 1998; Su et al., 2004). All eGFP-ZtTub2-labelled structures disappeared when cells were treated with benomyl (Fig. 3D), which is known to depolymerise specifically fungal microtubules (Fuchs et al., 2005; Sims et al., 1969), including those in *Z. tritici* (Bowler et al., 2010). These results confirm that the fusion protein eGFP-ZtTub2 incorporates into microtubules in *Z. tritici.*

**Fig. 4:**
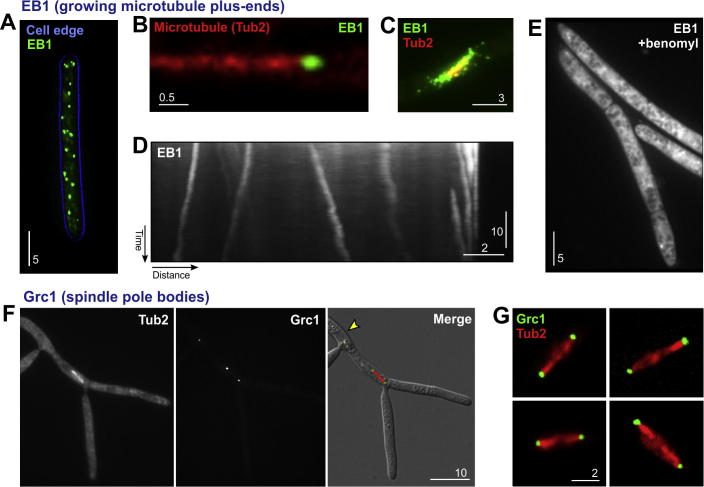
Microtubule plus- and minus-ends in *Z. tritici*. (A) Maximum projection of an image stack, showing all microtubule plus-ends, labelled with EB1-eGFP (green) in a yeast-like cell of *Z. tritici*. The cell edge is indicated by a blue-outline. Bar represents 5 μm. (B) Co-visualization of EB1-eGFP (green) and mCherry-Tub2 (red). Indeed, the putative plus-end binding protein binds to growing interphase microtubules in *Z. tritici*. Bar represents 0.5 μm. (C) Co-visualization of EB1-eGFP (green) and mCherry-Tub2 (red) in a mitotic spindle. Due to the dynamic nature of the spindle, numerous of EB1-eGFP signals concentrate around the spindle. Note that spindle microtubules appear yellow due to overlay with the green EB1-eGFP signals. Bar represents 3 μm. (D) Kymograph showing motility of EB1-eGFP signals in *Z. tritici*. Moving signals are represented by diagonal lines, whereas stationary signals are provide vertical lines. Note that EB1 labels only growing plus-ends. Bars represent 10 s and 2 μm. (E) EB1-eGFP after disruption of microtubules by 300 μM benomyl for 45 min, a reagent that prevents tubulin polymerization (Straube et al., 2003). No distinct “dots” are visible anymore, further confirming that EB1-eGFP labels microtubule plus-ends in *Z. tritici*. Bar represents 5 μm. (F) Co-visualization of mCherry-Tub2 and Grc1-eGFP demonstrates that the minus-end marker marks the spindle-pole bodies of the mitotic spindle. Here, microtubules get nucleated, confirming that Grc1-eGFP labels microtubule minus-ends. Note that cytoplasmic microtubule minus-ends are not visible, which is most likely due to the low number of Grc1-eGFP molecules binding to the γ-tubulin ring complex of individual interphase microtubules. Bar represents 10 μm. (G) Several examples of mitotic spindles, labelled with mCHerry-Tub2 (red) and Grc1-eGFP (green). Bar represents 2 μm.

We next set out to investigate the localization and dynamic behaviour of the putative microtubule plus-end binding protein ZtEB1. The fluorescent fusion protein ZtEB1-eGFP localized in comet-like spots throughout the entire cell (Fig. 4A, a yeast-like cell is shown; note that the image is a maximum projection of several planes, showing all ZtEB1-eGFP signals in the cell). Co-visualization of ZtEB1-eGFP and mCherry-labelled microtubules confirmed that the marker bound to microtubule ends (Fig. 4B; please note that mCherry-Tub2 labelling is much more weak than eGFP-Tub2 labelling and thus not recommended to visualize the entire microtubule array). In addition, ZtEB1-eGFP was concentrated along the fungal mitotic spindle, where highly dynamic microtubules are concentrated (Fig. 4C), and EB1-like protein is abundant (Straube et al., 2003). In interphase, ZtEB1-eGFP signals showed slow motility (Fig. 4D; kymograph shows moving signals as diagonal lines) at 5.3 ± 2.6 μm/min (*n* = 30). This velocity corresponds well with the microtubule polymerization velocities in other fungi (Drummond and Cross, 2000; Steinberg et al., 2001; Straube et al., 2003), confirming that ZtEB1-eGFP is localizing to the dynamic microtubule plus-end. Finally, we treated IPO323_HEB1eGFP with 300 μM benomyl for 45 min. The disassembly of microtubules distributed ZtEB1-eGFP in the cytoplasm (Fig. 4E), again arguing that the fusion protein binds to intact microtubules. We conclude that ZtEB1-eGFP is a useful marker for studying microtubule organization and dynamics in *Z. tritici*.

Finally, we investigated the cellular localization of the putative microtubule minus-end binding protein ZtGrc1-eGFP. In interphase cells, the signal intensity was very low and only a single fluorescent dot was visible (Fig. 4F, arrowhead). However, in mitotic cells, identified by the expression of mCherry-Tub2, two signals were visible (Fig. 4F). These were located at the poles of the mitotic spindle (Fig. 4F and G), where spindle microtubules are nucleated and γ-tubulin ring complex pr oteins are concentrated (Horio et al., 1991; Oakley et al., 1990; Prigozhina et al., 2001; Straube et al., 2003; Toya et al., 2007; Vardy and Toda, 2000). Thus, ZtGrc1-GFP localizes to sites of microtubule formation, which confirms its location at microtubule minus-ends.

### 3.7 Vectors for ectopic integration of all markers with alternative antibiotic resistant cassettes

Finally, we constructed a set of vectors that carried a second resistance cassette. This was done to provide the opportunity to combine any of the vectors with other constructs or fluorescently labelled components. Firstly, we generated vector pHeGFPTub2, designed for random ectopic integration of the eGFP-Tub2 fusion construct (Fig. 2E). This vector was derived from the vector pCeGFPTub2 (described above, Fig. 2A) and share most features, including *A. tumefaciens* -mediated transformation capacity and the capability to be used in yeast recombination-based cloning. It should be noted that this vector contains the *sdi1* downstream sequence (*sdi1* left flank and terminator, Fig. 2E). This sequence is a remnant of the cloning procedure and not of any functional significance. Finally, we constructed two vectors, pCEB1eGFP and pCGrc1eGFP, for homologous integration of eGFP into the native *Zteb1* and *Ztgrc1* locus. These vectors carry the carboxin-resistance cassette (Fig. 2F) and share the majority of features to the described vectors pHEB1eGFP and pHGrc1eGFP (see above, Fig. 2B). The vectors pCeGFPTub2, pCEB1eGFP or pCGrc1eGFP allow transformation into strains that already contain another resistant cassette, such as the selectable genes *hph* (hygromycin phosphotransferase; hygromycin-resistant), *nptII* (neomycin phosphotransferase; G418-resistant) or *bar* (phosphinothricin acetyltransferase; Basta-resistant; see Kilaru and Steinberg, 2015, for more details).

## 4 Conclusion

The microtubule cytoskeleton performs essential roles in mitosis and interphase of fungal cells. It is, therefore, a target for fungicide development (Holloman et al., 1997). Here, we provide fluorescent markers to visualize entire microtubules, their dynamic plus-ends and their nucleation sites. We provide evidence for their specific localization to microtubules, using pharmaceutical experiments and *in vivo* dual-color imaging. These markers will be useful tools to understand the organization and dynamic behaviour of the microtubule cytoskeleton in *Z. tritici*. This knowledge, in combination with the various vectors we provide, will help to characterize mutant phenotypes in *Z. tritici*. In addition, our tools will inform mode of action studies on new anti-fungals, aiming to develop novel control strategies for *Z. tritici* blotch on wheat.

## Figures and Tables

**Table 1:** Primers used in this study.

Primer name	Direction	Sequence (5′ to 3′)^a^
SK-Sep-10	Sense	*TGGCAGGATATATTGTGGTGTAAACAAATT*GACCTTCCACATCTACCGATGG
SK-Sep-11	Antisense	*ATTCAGAATGGTGAGGCATCGGTACAAGCT*CATGCTGTTGTTGAGTGCGTCC
SK-Sep-12	Sense	*AGCTTGTACCGATGCCTCACCATTCTGAAT* TGCTCAAGGACCTGCCCCAAG
SK-Sep-13	Antisense	CTTCCGTCGATTTCGAGACAGC
SK-Sep-14	Sense	*CATTTGCGGCTGTCTCGAAATCGACGGAAG*GCAGTCGACGCCAGATGATGG
SK-Sep-15	Antisense	*GGTGAACAGCTCCTCGCCCTTGCTCACCAT*GGCGATGGTGGTATGCGGATG
SK-Sep-16	Sense	ATGGTGAGCAAGGGCGAGGAG
SK-Sep-17	Antisense	CTTGTACAGCTCGTCCATGCCG
SK-Sep-18	Sense	*ATCACTCTCGGCATGGACGAGCTGTACAAG*ATGCGTGAAGTCATCTCCTTGAAC
SK-Sep-19	Antisense	GAGGAGTCGACAGCCAAGCTC
SK-Sep-25	Sense	*CTCTCATAAGAGCTTGGCTGTCGACTCCTC*ACATTTTACAACATACTCAAGTCTG
SK-Sep-26	Antisense	*TAAACGCTCTTTTCTCTTAGGTTTACCCGC*GTTGAAGTTCTGCGTCGGATCC
SK-Sep-37	Sense	*TAATTCCTAGGCCACCATGTTGGGCCCGGC*GCGCCAACCTGGATCCGTGG
SK-Sep-38	Antisense	*GGTGAACAGCTCCTCGCCCTTGCTCACCAT*ACTAAATGCGCCGACGACGCC
SK-Sep-47	Antisense	GGCGATGGTGGTATGCGGATG
SK-Sep-48	Sense	*TAATTCCTAGGCCACCATGTTGGGCCCGGC*CAGCATCTTCCTCGATGTGCCC
SK-Sep-49	Antisense	*GGTGAACAGCTCCTCGCCCTTGCTCACCAT*AAACGTCTCCTCCTCGCCCGC
SK-Sep-51	Antisense	*TAAACGCTCTTTTCTCTTAGGTTTACCCGC*AACGGAGGACTGGGCTTGAAGC
SK-Sep-53	Antisense	*TAAACGCTCTTTTCTCTTAGGTTTACCCGC*CGAGATGTTGGAGTCCAGGATC
SK-Sep-60	Antisense	*ATCGAAGGTCTCTGCTCGCCGCTCGGTAGC*CGATGAATTCTCATGTTTGACAGC
SK-Sep-83	Sense	*CATCACTCACATCCGCATACCACCATCGCC*ATGGTGAGCAAGGGCGAGGAGG
SK-Sep-92	Antisense	*CGTACCGTTCAAGGAGATGACTTCACGCAT*CTTGTACAGCTCGTCCATGCCGC
SK-Sep-96	Sense	GCTACCGAGCGGCGAGCAGA
SK-Sep-97	Antisense	CTTCCGTCGATTTCGAGACAGC
SK-Sep-99	Sense	*CATTTGCGGCTGTCTCGAAATCGACGGAAG*TGAGACGCTGTTCATAACAATGTC
SK-Sep-100	Sense	*CATTTGCGGCTGTCTCGAAATCGACGGAAG*TACCATGATTTGCTTGAGCGGCTC
SK-Sep-128	Sense	*CTCTCATAAGAGCTTGGCTGTCGACTCCTC*GAATTCGAGCTCGGTACCCAACT
SK-Sep-129	Antisense	*CTTTTCTCTTAGGTTTACCCGCGTTGAAGT*GCGTTAACACTAGTCAGATCTACC
SK-Sep-130	Sense	*TGATAAGCTGTCAAACATGAGAATTCATCG*GAATTCGAGCTCGGTACCCAACT
SK-Sep-132	Antisense	*AATGATGACATTGTTATGAACAGCGTCTCA*GCGTTAACACTAGTCAGATCTACC
SK-Sep-133	Antisense	*ACAAATGAGCCGCTCAAGCAAATCATGGTA*GCGTTAACACTAGTCAGATCTACC

a *Italics* indicate part of the primer that is complementary with another DNA fragment, to be ligated by homologous recombination in *S. cerevisiae.*

## References

[b0005] Abenza J.F., Pantazopoulou A., Rodriguez J.M., Galindo A., Penalva M.A. (2009). Long-distance movement of *Aspergillus nidulans* early endosomes on microtubule tracks. Traffic.

[b0015] Bielska E., Higuchi Y., Schuster M., Steinberg N., Kilaru S., Talbot N.J., Steinberg G. (2014). Long-distance endosome trafficking drives fungal effector production during plant infection. Nat. Commun..

[b0020] Bowler J., Scott E., Tailor R., Scalliet G., Ray J., Csukai M. (2010). New capabilities for *Mycosphaerella graminicola* research. Mol. Plant Pathol..

[b0025] Boyle J.S., Lew A.M. (1995). An inexpensive alternative to glassmilk for DNA purification. Trends Genet..

[b0030] Clemons G.P., Sisler H.D. (1971). Localization of the site of action of a fungitoxic benomyl derivative. Pestic. Biochem. Physiol..

[b0035] Desai A., Mitchison T.J. (1997). Microtubule polymerization dynamics. Ann. Rev. Cell Dev. Biol..

[b0040] Drummond D.R., Cross R.A. (2000). Dynamics of interphase microtubules in *Schizosaccharomyces pombe*. Curr. Biol..

[b0045] Egan M.J., McClintock M.A., Reck-Peterson S.L. (2012). Microtubule-based transport in filamentous fungi. Curr. Opin. Microbiol..

[b0055] Fink G., Steinberg G. (2006). Dynein-dependent motility of microtubules and nucleation sites supports polarization of the tubulin array in the fungus *Ustilago maydis*. Mol. Biol. Cell.

[b0050] Fink, G., Schuchardt, I., Colombelli, J., Stelzer, E., Steinberg, G., 2006. Dynein-mediated pulling forces drive rapid mitotic spindle elongation in *Ustilago maydis*. EMBO J.25, pp. 4897–4908. 10.1038/sj.emboj.7601354 PMC1618106 17024185

[b0060] Fuchs U., Manns I., Steinberg G. (2005). Microtubules are dispensable for the initial pathogenic development but required for long-distance hyphal growth in the corn smut fungus *Ustilago maydis*. Mol. Biol. Cell.

[b0065] Goodwin S.B., M’Barek S.B., Dhillon B., Wittenberg A.H., Crane C.F., Hane J.K. (2011). Finished genome of the fungal wheat pathogen *Mycosphaerella graminicola* reveals dispensome structure, chromosome plasticity, and stealth pathogenesis. PLoS Genet..

[b0070] Higuchi Y., Ashwin P., Roger Y., Steinberg G. (2014). Early endosome motility spatially organizes polysome distribution. J. Cell Biol..

[b0075] Hirokawa N., Takemura R. (2004). Molecular motors in neuronal development, intracellular transport and diseases. Curr. Opin. Neurobiol..

[b0080] Hoffman C.S., Winston F. (1987). A ten-minute DNA preparation from yeast efficiently releases autonomous plasmids for transformation of *Escherichia coli*. Gene.

[b0085] Holloman, D.W., Butters, J.A., Barker, H., 1997. Tubulins, a target for anti-fungal agents., London, United Kingdom, Royal Society of Chemistry.

[b0090] Holsters M., de Waele D., Depicker A., Messens E., van Montagu M., Schell J. (1978). Transfection and transformation of *Agrobacterium tumefaciens*. Mol. Gen. Genet..

[b0095] Hood E., Gelvin S.B., Melchers L., Hoekema A. (1993). New *Agrobacterium* helper plasmids for gene transter to plants. Transgen. Res..

[b0100] Horio T., Oakley B.R. (2005). The role of microtubules in rapid hyphal tip growth of *Aspergillus nidulans*. Mol. Biol. Cell.

[b0105] Horio T., Uzawa S., Jung M.K., Oakley B.R., Tanaka K., Yanagida M. (1991). The fission yeast γ-tubulin is essential for mitosis and is localized at microtubule organizing centers. J. Cell Sci..

[b0110] Job D., Valiron O., Oakley B. (2003). Microtubule nucleation. Curr. Opin. Cell Biol..

[b0115] Jung M.K., May G.S., Oakley B.R. (1998). Mitosis in wild-type and β-tubulin mutant strains of *Aspergillus nidulans*. Fungal Genet. Biol..

[b0120] Kema G.H.J., van Silfhout C.H. (1997). Genetic variation for virulence and resistance in the wheat-*Mycosphaerella graminicola* pathosystem. III. Comparative seedling and adult plant experiments. Phytopathology.

[b0125] Kilaru, S., Steinberg, G., 2015. Yeast recombination-based cloning as an efficient way of constructing vectors for *Zymoseptoria tritici*. Fungal Genet. Biol 79, 76–83. 10.1016/j.fgb.2015.03.017 PMC4502459 26092792

[b0130] Kilaru, S., Schuster, M., Latz, M., Das Gupta, S., Steinberg, N., Fones, H., Gurr S., Talbot, N.J., Steinberg, G., 2015a. A gene locus for targeted ectopic gene integration in *Zymoseptoria tritici*. Fungal Genet. Biol 79, 118–124. 10.1016/j.fgb.2015.03.018 PMC4502457 26092798

[b0135] Kilaru, S., Schuster, M., Latz, M., Guo, M., Steinberg, G., 2015b. Fluorescent markers of the endocytic pathway in *Zymoseptoria tritici*. Fungal Genet. Biol 79, 150–157 10.1016/j.fgb.2015.03.019 PMC4502447 26092801

[b0140] Knop M., Schiebel E. (1997). Spc98p and Spc97p of the yeast γ-tubulin complex mediate binding to the spindle pole body via their interaction with Spc110p. EMBO J..

[b0145] Konzack S., Rischitor P.E., Enke C., Fischer R. (2005). The role of the kinesin motor KipA in microtubule organization and polarized growth of *Aspergillus nidulans*. Mol. Biol. Cell.

[b0150] Lenz J.H., Schuchardt I., Straube A., Steinberg G. (2006). A dynein loading zone for retrograde endosome motility at microtubule plus-ends. EMBO J..

[b0155] Martin M.A., Osmani S.A., Oakley B.R. (1997). The role of γ-tubulin in mitotic spindle formation and cell cycle progression in *Aspergillus nidulans*. J. Cell Sci..

[b0160] Moritz M., Braunfeld M.B., Sedat J.W., Alberts B., Agard D.A. (1995). Microtubule nucleation by γ-tubulin-containing rings in the centrosome. Nature.

[b0165] Oakley C.E., Oakley B.R. (1989). Identification of γ-tubulin, a new member of the tubulin superfamily encoded by *mipA* gene of *Aspergillus nidulans*. Nature.

[b0170] Oakley B.R., Oakley C.E., Yoon Y., Jung M.K. (1990). Γ-tubulin is a component of the spindle pole body that is essential for microtubule function in *Aspergillus nidulans*. Cell.

[b0010] Baumann S., Pohlmann T., Jungbluth M., Brachmann A., Feldbrügge M. (2012). Kinesin-3 and dynein mediate microtubule-dependent co-transport of mRNPs and endosomes. J. Cell Sci..

[b0175] Pantazopoulou A., Pinar M., Xiang X., Peñalva M.A. (2014). Maturation of late Golgi cisternae into RabE^RAB11^ exocytic post-Golgi carriers visualized in vivo. Mol. Biol. Cell.

[b0180] Prigozhina N.L., Walker R.A., Oakley C.E., Oakley B.R. (2001). Γ-tubulin and the C-terminal motor domain kinesin-like protein, KLPA, function in the establishment of spindle bipolarity in *Aspergillus nidulans*. Mol. Biol. Cell.

[b0185] Raymond C.K., Pownder T.A., Sexson S.L. (1999). General method for plasmid construction using homologous recombination. BioTechniques.

[b0190] Riehlman T.D., Olmsted Z.T., Branca C.N., Winnie A.M., Seo L., Cruz L.O., Paluh J.L. (2013). Functional replacement of fission yeast γ-tubulin small complex proteins Alp4 and Alp6 by human GCP2 and GCP3. J. Cell Sci..

[b0195] Rohel E.A., Payne A.C., Hall L., Barker H., Butters J., Hollomon D.W. (1998). Isolation and characterization of α-tubulin genes from *Septoria tritici* and *Rhynchosporium secalis*, and comparative analyzis of fungal α-tubulin sequences. Cell Motil. Cytoskeleton.

[b0200] Sambrook J., Russell D.W. (2001). Molecular cloning.

[b0205] Schuchardt I., Assmann D., Thines E., Schuberth C., Steinberg G. (2005). Myosin-V, kinesin-1, and kinesin-3 cooperate in hyphal growth of the fungus *Ustilago maydis*. Mol. Biol. Cell.

[b0210] Schuster M., Kilaru S., Fink G., Collemare J., Roger Y., Steinberg G. (2011). Kinesin-3 and dynein cooperate in long-range retrograde endosome motility along a nonuniform microtubule array. Mol. Biol. Cell.

[b0215] Schuster M., Treitschke S., Kilaru S., Molloy J., Harmer N.J., Steinberg G. (2012). Myosin-5, kinesin-1 and myosin-17 cooperate in secretion of fungal chitin synthase. EMBO J..

[b0220] Seidel C., Moreno-Velasquez S.D., Riquelme M., Fischer R. (2013). Neurospora crassa NKIN2, a kinesin-3 motor, transports early endosomes and is required for polarized growth. Eukaryot. Cell.

[b0225] Sims J.J., Mee H., Erwin D.C. (1969). Methyl 2-benzimidazolecarbamate, a fungitoxic compound isolated from cotton plants treated with methyl 1-(butylearbamoyl,-2-benzimidazolecarbamate, benomyl. Phytopathology.

[b0230] Steinberg G. (2014). Endocytosis and early endosome motility in filamentous fungi. Curr. Opin. Microbiol..

[b0235] Steinberg G., Wedlich-Söldner R., Brill M., Schulz I. (2001). Microtubules in the fungal pathogen *Ustilago maydis* are highly dynamic and determine cell polarity. J. Cell Sci..

[b0240] Straube A., Brill M., Oakley B.R., Horio T., Steinberg G. (2003). Microtubule organization requires cell cycle-dependent nucleation at dispersed cytoplasmic sites, polar and perinuclear microtubule organizing centers in the plant pathogen Ustilago maydis. Mol. Biol. Cell.

[b0245] Su W.Q., Li S.H., Oakley B.R., Xiang X. (2004). Dual-color Imaging of nuclear division and mitotic spindle elongation in live cells of *Aspergillus nidulans*. Eukaryot Cell.

[b0250] Szewczyk E., Oakley B.R. (2011). Microtubule dynamics in mitosis in *Aspergillus nidulans*. Fungal Genet. Biol..

[b0255] Tamura K., Peterson D., Peterson N., Stecher G., Nei M., Kumar S. (2011). MEGA5, molecular evolutionary genetics analyzis using maximum likelihood, evolutionary distance, and maximum parsimony methods. Mol. Biol. Evol..

[b0260] Tang X., Halleck M.S., Schlegel R.A., Williamson P. (1996). A subfamily of P-type ATPases with aminophospholipid transporting activity. Science.

[b0265] Teixido-Travesa N., Roig J., Lüders J. (2012). The where, when and how of microtubule nucleation - one ring to rule them all. J. Cell Sci..

[b0270] Toya M., Sato M., Haselmann U., Asakawa K., Brunner D., Antony C., Toda T. (2007). Γ-tubulin complex-mediated anchoring of spindle microtubules to spindle-pole bodies requires Msd1 in fission yeast. Nat. Cell Biol..

[b0275] Vale R.D. (2003). The molecular motor toolbox for intracellular transport. Cell.

[b0315] Vardy L., Toda T. (2000). The fission yeast γ-tubulin complex is required in G(1, phase and is a component of the spindle assembly checkpoint. EMBO J..

[b0285] Wedlich-Söldner R., Bölker M., Kahmann R., Steinberg G. (2000). A putative endosomal t-SNARE links exo- and endocytosis in the phytopathogenic fungus *Ustilago maydis*. EMBO J..

[b0320] Wedlich-Söldner R., Straube A., Friedrich M.W., Steinberg G. (2002). A balance of KIF1A-like kinesin and dynein organizes early endosomes in the fungus *Ustilago maydis*. EMBO J..

[b0295] Wiese C., Zheng Y. (2006). Microtubule nucleation, γ-tubulin and beyond. J. Cell Sci..

[b0300] Xiang X., Plamann M. (2003). Cytoskeleton and motor proteins in filamentous fungi. Curr. Opin. Microbiol..

[b0305] Zheng Y., Jung M.K., Oakley B.R. (1991). Γ-tubulin is present in *Drosophila melanogaster* and *Homo sapiens* and is associated with the centrosome. Cell.

[b0310] Zwiers L.H., De Waard M.A. (2001). Efficient *Agrobacterium tumefaciens*-mediated gene disruption in the phytopathogen *Mycosphaerella graminicola*. Curr. Genet..

